# Adoption of Lutetium-^177^ PSMA radioligand therapy for metastatic castration resistant prostate cancer: a total population analysis in Germany from 2016 to 2020

**DOI:** 10.1007/s00259-023-06139-x

**Published:** 2023-02-24

**Authors:** Luka Flegar, Smita George Thoduka, Damiano Librizzi, Markus Luster, Aristeidis Zacharis, Hendrik Heers, Nicole Eisenmenger, Hojjat Ahmadzadehfar, Matthias Eiber, Wolfgang Weber, Christer Groeben, Johannes Huber

**Affiliations:** 1 Department of Urology, Philipps-University Marburg, Baldinger Street, 35043 Marburg, Germany; 2 Department of Nuclear Medicine, Philipps-University Marburg, Baldinger Street, 35043 Marburg, Germany; 3 Reimbursement Institute, Hürth, Germany; 4 Department of Nuclear Medicine, Klinikum Westfalen GmbH, Dortmund, Germany; 5 Department of Nuclear Medicine, Technische Universität Munich, Munich, Germany

**Keywords:** Prostate cancer, mCRPC, Lutetium-^177^ PSMA (^177^Lu-PSMA), Radioligand therapy, Health services research, Trends

## Abstract

**Purpose:**

This study is to investigate the adoption and current trends of Lutetium-^177^ PSMA RLT for mCRPC in Germany.

**Methods:**

We analyzed data from the reimbursement.INFO tool based on German hospitals’ quality reports for Lutetium-^177^ PSMA RLT from 2016 to 2020 and from the nationwide German hospital billing database (Destatis) for general therapy with open radionuclides in combination with prostate cancer from 2006 to 2020. For validation of these billing data, we included the ^177^Lu-PSMA RLT cycles from two participating institutions from 2016 to 2020. For detection of trends over time we applied linear regression models.

**Results:**

General therapy with open radionuclides increased from 2006 to 2020. We identified a total of 12,553 ^177^Lu-PSMA RLT cycles. The number of ^177^Lu-PSMA RLTs steadily increased from a total of 1026 therapies in 2016 to 3328 therapies in 2020 (+ 576 RLT/year; *p* < 0.005). In 2016, 25 departments of nuclear medicine offered this treatment, which increased to 44 nuclear medicine departments in 2020. In 2016, 16% of nuclear medicine departments (4/25) performed more than 100 ^177^Lu-PSMA RLTs, which increased to 36% (16/44) in 2020 (*p* < 0.005). In 2016, 88% (22/25) of 177Lu-PSMA RLTs were performed at a university hospital, which decreased to 70% (31/44) in 2020. The proportion of patients older than 65 years receiving ^177^Lu-PSMA RLT increased from 78% in 2016 to 81% in 2020.

**Conclusion:**

Treatment of mCRPC with ^177^Lu-PSMA RLT has been rapidly increasing in Germany in the recent years providing an additional therapy option. This development is remarkable, because of outstanding formal EMA approval.

**Supplementary Information:**

The online version contains supplementary material available at 10.1007/s00259-023-06139-x.

## Introduction

Since prostate cancer cells are uniquely connected to the androgen pathway and stimulated by it, therapy for metastatic PCa includes suppressing testosterone production through androgen deprivation therapy (ADT) or surgery [1]. However, nearly all the patients on ADT will develop resistance and reach metastatic castration-resistant prostate cancer (mCRPC) stage eventually [2, 3].

Throughout the last decade, multiple new systemic treatment options have become available for patients with mCRPC [4, 5]. Among these, radioligand therapy (RLT) with ^177^Lutetium (Lu) prostate-specific membrane antigen (PSMA) is a relatively new treatment option which has gained a significant popularity in recent years for patients with mCRPC who progressed after chemotherapy and new hormonal agents [6].

Several studies showed a great response and prolonged survival combined with a low rate of adverse events [4, 7]. Rahbar et al. presented in 2017 their initial experience and results of a German multicenter study, which analyzed ^177^Lu-PSMA-617 RLT in advanced PCa [8]. They were able to demonstrate favorable safety and high efficacy exceeding those of other third-line systemic therapies in mCRPC patients. A further working group from Australia presented early results on patients with mCRPC receiving ^177^Lu-PSMA RLT [9]. The conducted meta-analysis showed that approximately two-thirds of patients had a biochemical response to ^177^Lu-PSMA RLT [9]. The therapy with Lutetium-^177^ is especially effective, since it is particularly precise and targeted. Lutetium-^177^ PSMA-617 and ^177^Lu-PSMA I&T are ligands that are coupled with radioactive Lutetium-^177^ [10, 11]. Therefore, they can dock specifically to PSMA, which is a glycoprotein that is found on the majority of all prostate cancer cells [12]. In the next step, the active substance is taken up into the cell interior of the cancer cell. Consequently, the tumor cells accumulate the lethal dose of radiation leading to apoptosis of the cancer cells, thus sparing adjacent normal organs and tissues [10, 13].

PSMA ^177^Lutetium RLT was first clinically introduced in Germany at a few selected nuclear medicine centers in 2015 [8]. The great success of PSMA ^177^Lutetium RLT started with PSMA positron emission tomography (PET) imaging for metastatic PCa [14]. Hallmark of PSMA PET for diagnostics is the accuracy for localization of initial or recurrent prostate cancer (PCa) [15]. Consequently, in the era of theranostics, PSMA-targeted therapies with alpha- or beta-emitters were introduced [2, 16].

A recent study from 2021 showed evidence that RLT with ^177^Lu-PSMA-617 delayed the progression for patients with mCRPC who have been treated with an androgen-receptor inhibitor and taxane-based chemotherapy previously and also significantly prolonged overall survival, which opened the way for PSMA ^177^Lutetium RLT approval by the Food and Drug Administration (FDA) in March 2022 in the USA [4].

Since Lutetium-^177^ PSMA-617 RLT is more commonly applied in clinical routine for patients with metastatic PCa and approval by the European Medicines Agency (EMA) is expected later this year, the aim of the present study was to investigate the adoption of this treatment in Germany from 2016 to 2020.

## Patients and methods

### Database

We analyzed population-based data from the German hospital quality reports between 2016 and 2020. Table 1 provides an overview of the queried databases. We described data extraction and cohort identification in previous studies [17, 18]. By using the reimbursement.INFO tool (Reimbursement Institute, Hürth, Germany) we were able to analyze the annual case cycles of ^177^Lu-PSMA RLTs on an institutional level. The German hospitals have been required by law to provide quality reports since 2005. The quality reports contain information on diagnoses and treatments, the frequency of treatment, staffing levels, the number of certain complications, and accessibility. ^177^Lu-PSMA RLT was defined by operation and procedure code (OPS code) “8–530.d0.” The OPS code “8–530.d0” is available since 2016 for reimbursement of this therapy and applies for in-patient treatment of patients irrespective of their insurance status. We classified hospitals and departments into university and non-university hospitals according to the quality reports.

**Table 1:** Overview of the queried databases

Data source	Nationwide hospital billing database of the German Federal Statistical Office (Destatis database)	German hospitals’ quality reports (reimbursement.INFO tool)
Data details	• Age and gender • Diagnosis code • Type of surgery and approach • Hospital characteristics (teaching status, size, annual surgery caseload, approaches for surgery)	• Age and gender • Type of surgery • Hospital characteristics (teaching status, annual surgery caseload) • Geographical localization of respective hospitals
Data query option	• Combination of OPS- and DRG code possible	• Only OPS- or DRG code
Number of patients	13.702	12.553
Proportion of the country	100%	100%
Included years	2006–2020	2016–2020

Furthermore, we included data from the German Federal Statistical Office (Destatis) between 2006 and 2020. The Destatis entertains a nationwide billing database, and the advantage of this database is the possibility of combining OPS and ICD codes during the data query. We identified all cases with a diagnosis of PCa (ICD-10: C.61) in combination with procedural codes for ^177^Lu-PSMA RLT (OPS code “8–530.d0”). Additionally, we analyzed the less specific code “therapy with open radionuclides” (OPS code “8–530”) in combination with PCa. In-hospital mortality, blood transfusions, and length of hospital stay (LOS) for ^177^Lu-PSMA RLT were evaluated.

### Development of coding over time

During our analysis, we observed a discrepancy between the databases in coding of ^177^Lu-PSMA RLT. Therefore, we further analyzed all patients who received ^177^Lu-PSMA RLT at two German institutions (TU Munich and University Hospital Marburg) between 2016 and 2020. This analysis of the two centers confirmed our initial suspicion of two principal ways of coding. Especially in 2016, the specific code (OPS code “8–530.d0”) was less frequently used for ^177^Lu-PSMA RLT. In the subsequent years, the specific code was much more applied for coding of ^177^Lu-PSMA RLT. Therefore, we supplemented the evaluation of quality reports with a combined query of diagnosis and OPS in the Destatis database.

### Data protection and ethics statement

This study was conducted in accordance with the Declaration of Helsinki in its latest version. For data protection reasons, within the quality reports, the diagnostics (ICD) data or intervention numbers (OPS) with a number of ≤3 does not indicate the actual number, but the number 1. All data used are anonymized, so no further ethics committee approval was required. This article does not contain any studies with animals performed by any of the authors. Further, a written informed consent was not needed. We followed the REporting of studies Conducted using Observational Routinely collected health Data statement (RECORD) [19].

### Statistics

Linear regression models to detect trends over time were performed. Data were presented by absolute and relative frequencies, standard deviation, and mean. We defined *p* < 0.05 to indicate statistical significance. SPSS 27.0 (IBM corp., Armonk, NY, USA) was used for our statistical analysis. The maps were created using “EasyMap 11.1 Standard Edition” (Lutum + Tappert DVBeratung GmbH, Bonn, Germany).

## Results

**Fig. 1:**
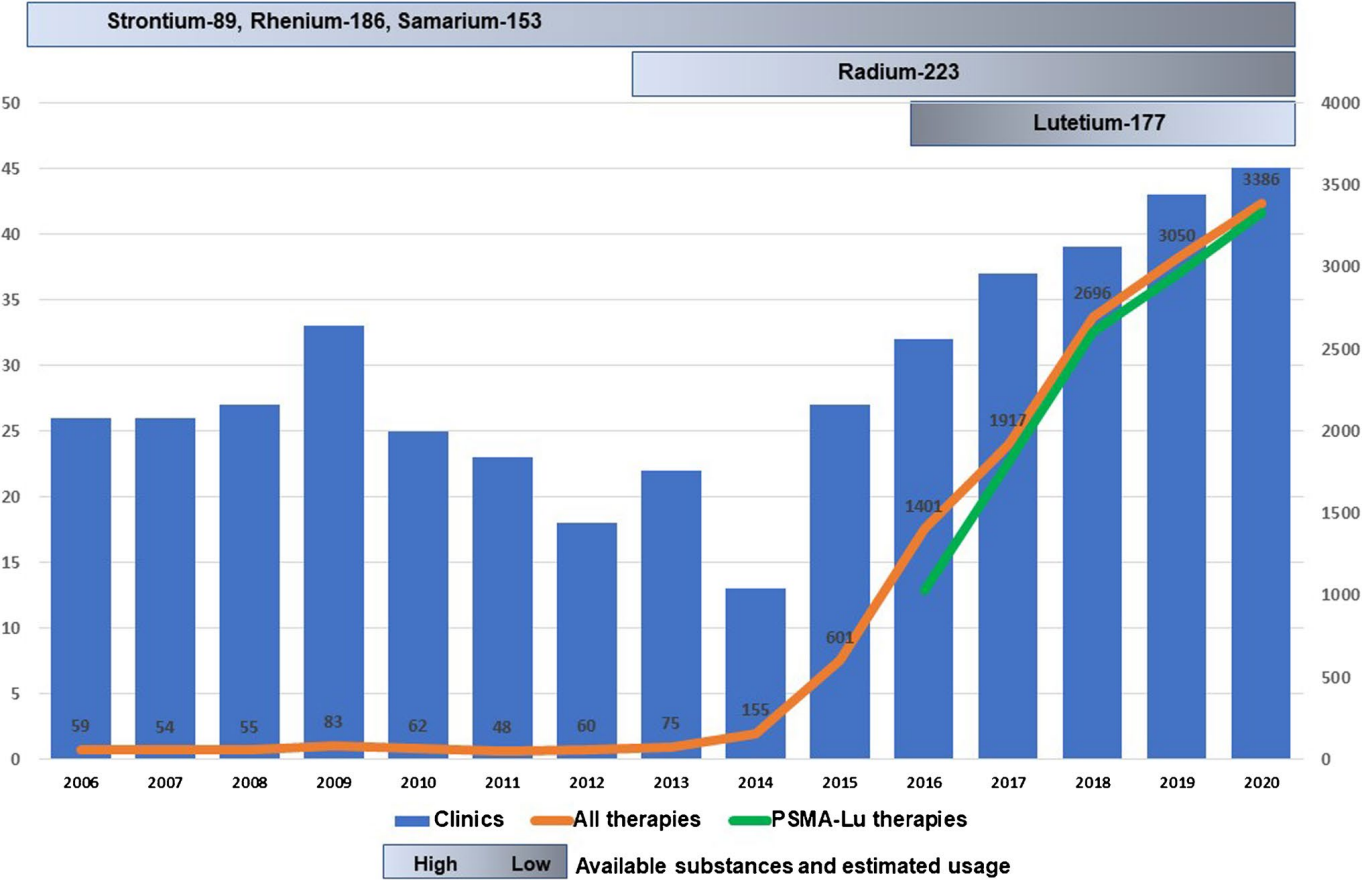
The orange line indicates the total number of all performed therapies per year. The green line indicates the total number of performed ^177^Lu-PSMA RLT per year. The blue columns represent the number of clinics providing the therapy. The grey columns show the available substances chronologically and their estimated usage (Source: Destatis database)

We were able to include a total of 12553 ^177^Lu-PSMA RLT cycles between 2016 and 2020. Figure 1 represents the annual number of performed ^177^Lu-PSMA RLT and therapies with open radio nuclides in combination with PCa as well as the number of clinics providing the therapy from 2006 to 2020. The number of ^177^Lu-PSMA RLTs steadily increased by 224% from a total of 1026 therapies in 2016 to 3328 therapies in 2020 (+576 RLT/year; *p* < 0.005). Therapies with open radionuclides for PCa treatment stayed constant around 60 cases per year between 2006 and 2013 (*p* = 0.546). Since 2014, the therapies with open radio nuclides increased from 155 to 3386 cases in 2020 (+459 therapies/year; *p* < 0.005). The difference between clinics using the specific or non-specific code decreased from 7 clinics in 2016 to 1 in 2020. For all performed therapies, the difference between the two codes decreased from 375 to 58 cases in 2020.

Table 1 in the online supplement displays the caseload of ^177^Lu-PSMA RLT cycles for the years 2016 to 2020 from two further centers.

**Fig. 2:**
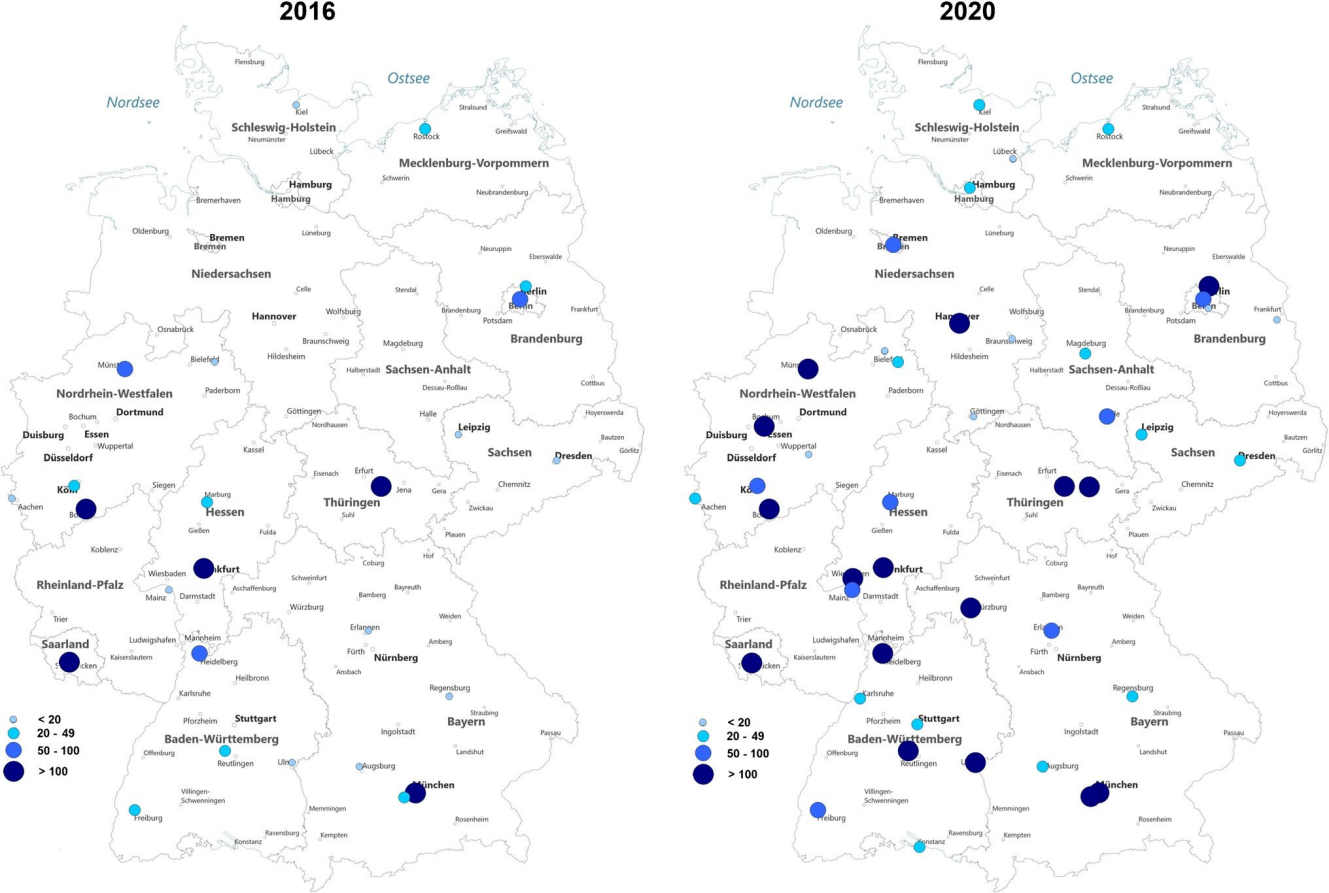
Overview of departments of nuclear medicine offering ^177^Lu-PSMA radioligand therapy in Germany in 2016 (left) and 2020 (right) (source: German hospitals’ quality reports corrected according to local case records for TU Munich in 2016)

In 2016, 25 departments of nuclear medicine offered the therapy, which increased to 44 nuclear medicine departments in 2020 (*p* < 0.005; +232%) (Fig. 1 in online supplement). Figure 2 provides an overview of nuclear medicine clinics in Germany offering 177Lu-PSMA RLT in 2016 and 2020. In 2016, 16% of nuclear medicine departments (4/25) performed more than 100 ^177^Lu-PSMA RLTs, which increased to 36% (16/44) in 2020 (*p* < 0.005).

**Fig. 3:**
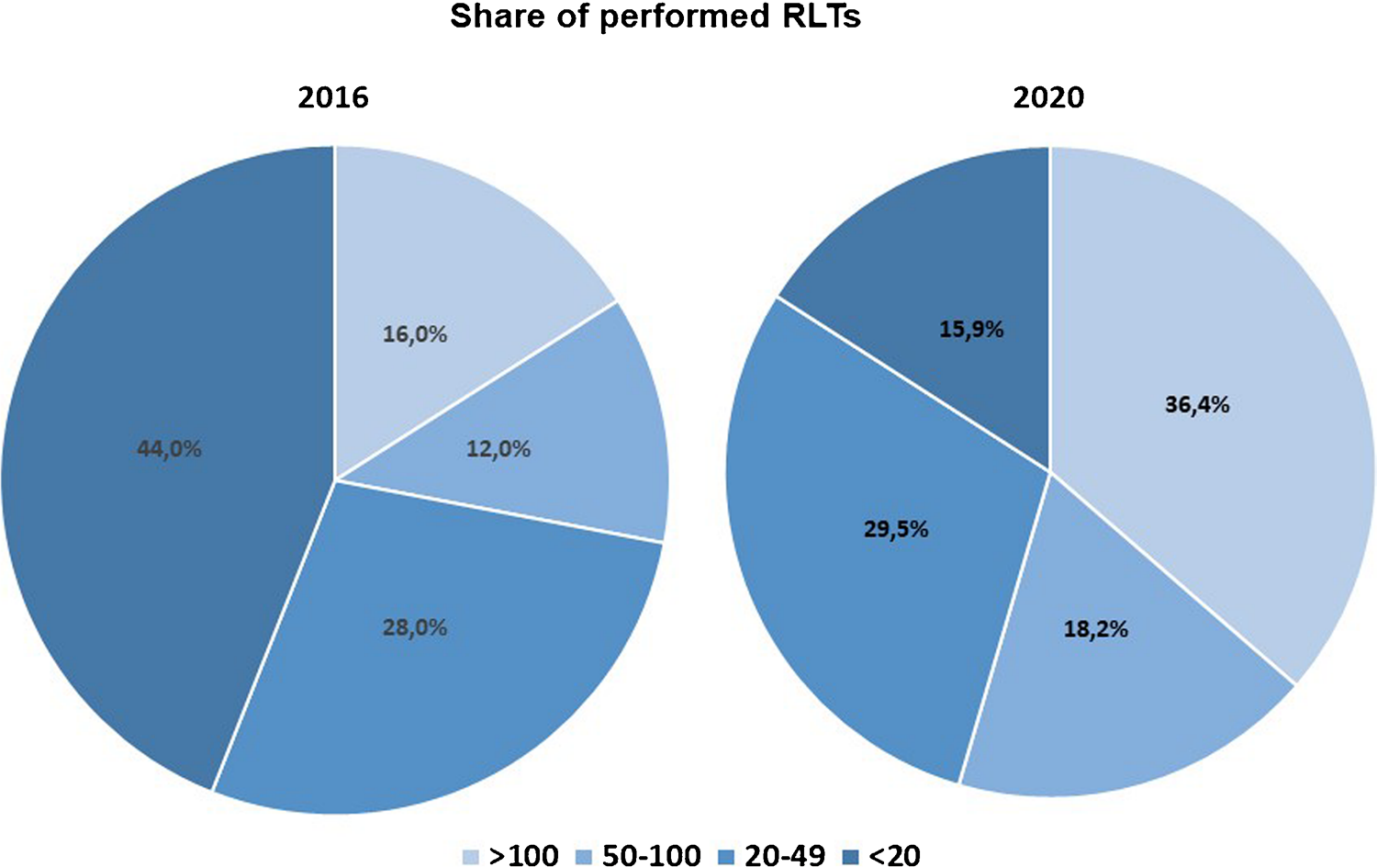
^177^Lu-PSMA RLTs performance in 2016 and 2020 by percentage according to performed cases per department (<20, 20–49, 50–100, >100) (source: German hospitals’ quality reports)

In 2016, 3 departments (12%) performed 50–100 ^177^Lu-PSMA RLTs, 7 departments (28%) performed 20–49 therapies, and 11 departments (44%) performed <20 therapies (*p* < 0.005). In 2020, 8 departments (18%) performed 50–100 177Lu-PSMA RLTs, 13 departments (30%) 20–49 therapies, and 7 departments (16%) performed <20 therapies (*p* < 0.005) (Fig. 3).

In 2016, 88% (22/25) of ^177^Lu-PSMA RLTs were performed at a university hospital, which decreased to 70% (31/44) in 2020. The procedure was exclusively performed in departments of nuclear medicine from 2016 to 2020.

**Fig. 4:**
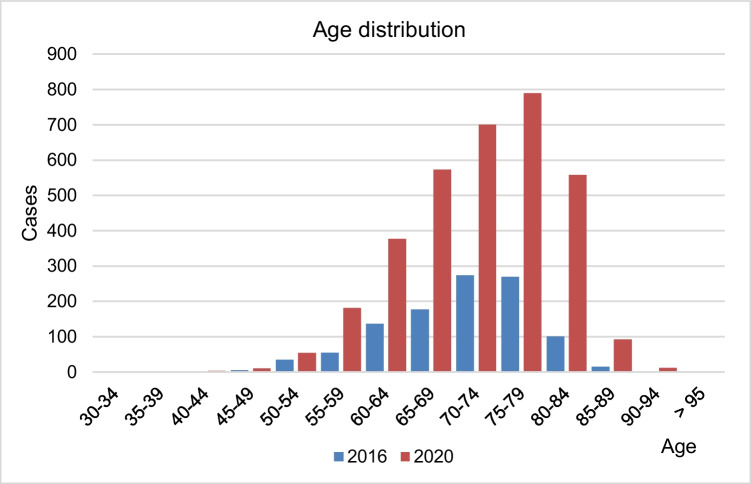
Age distribution in patients treated with 177Lu-PSMA radioligand therapy in 2016 (blue) and 2020 (red) (source: German hospitals’ quality reports)

Figure 4 shows the case numbers of received therapies stratified by age in 2016 and 2020. The proportion of patients older than 65 years receiving ^177^Lu-PSMA RLT increased from 78% in 2016 to 81% in 2020 (*p* < 0.005).

In-hospital mortality was 0.2% (15 out of 8389 cases) for all ^177^Lu-PSMA RLT between 2016 and 2019. The overall rate of blood transfusion after ^177^Lu-PSMA RLT was 4% (338 out of 8389 cases). The median LOS for ^177^Lu-PSMA RLT was 3 days (SD: 2.02).

## Discussion

This is internationally the first population-based study describing ^177^Lu-PSMA RLT adoption for mCRPC. We observed a steady increase of case numbers as well as from clinics offering the therapy. In 2020, a total of 44 German hospitals performed 3328 RLT cycles. It is important to consider that in Germany, a special situation exists in that the therapy was originally developed by the German Cancer Research Center and the University Hospital Heidelberg and therefore was reimbursed even without EMA approval [20]. However, due to several reasons, the development of ^177^Lu-PSMA-617 RLT did not follow the usual path of drug development [3]. The therapy was initially conducted as a palliative or near-last line of treatment before first formal randomized controlled trials were conducted [3, 21].

### Adoption of ^177^Lu-PSMA RLT

We noticed a significant increase of ^177^Lu-PSMA RLTs by 224% between 2016 and 2020. Rahbar et al. presented in October 2016 retrospective data on patients treated with PSMA ^177^Lutetium RLT under the compassionate-use program at 12 hospitals in Germany [8, 21]. Besides good tolerability and safety of the procedure, the authors were able to show high efficacy of ^177^Lu-PSMA RLT with an overall biochemical response rate of 45% after all therapy cycles, while 40% of patients already responded after a single cycle [8]. These very promising oncological results were a catalysator for broad adoption of ^177^Lu-PSMA RLT in Germany and later paved the way for the FDA to open the prospective phase 3 VISION study in the USA [21]. Our analysis showed a low in-hospital mortality rate of 0.2% proving that the safety of the therapy while a transfusion was only required in 4% of patients. Groener et al. showed hematologic safety of ^177^Lu-PSMA RLT in 2021 [22]. Our results showed, that the difference between clinics using the non-specific code and the specific code for reimbursement of ^177^Lu-PSMA RLT was highest in 2016 and then decreased to around <10%. This showed that with time clinics adapted their coding to the newly introduced specific code for ^177^Lu-PSMA RLT. We noticed a constant number of patients and clinics offering a therapy with open radionuclides for PCa between 2006 and 2013 which is most likely associated to palliative pain therapy with osteotropic radiopharmaceuticals as a treatment option for symptomatic skeletal metastases that cannot be adequately treated with drug pain therapy [23]. Since 2013, radium-233 therapy is also available for treatment of bone metastasis [23].

### Patient’s age, length of stay, and university hospital setting

Our results showed that ^177^Lu-PSMA RLT was increasingly applied in older patients. In 2020, 81% of patients receiving ^177^Lu-PSMA RLT were above 65 years of age. Gadot et al. described in their single-center study that the median age at first ^177^Lu-PSMA-617 treatment was 74.4 (range 56.6–91.9). Interestingly, an age above 77 years was associated with a significantly higher PSA response above 20% in univariate analysis [3]. Our analysis showed that most patients underwent three therapy cycles and the LOS was in general 3 days. In Germany, ^177^Lu-PSMA-617 treatment must be administered in an inpatient setting with a minimum 3 days hospital admission due to the patient-specific radiation dose, whereas in Australia according to Emmett et al., patients undergoing ^177^Lu-PSMA RLT with a standard 6–8 Gbq dose fall within the range which can be administered safely and legally within an outpatient setting [6]. In the present study, we observed that most ^177^Lu-PSMA RLTs were applied in a university hospital setting at nuclear medicine departments. This phenomenon may be initially connected to the novelty of the RLT as well as the limited availability of ^177^Lu-PSMA in the early applications [24]. In 2020, 70% of performed RLTs were administered at a university hospital out of which 20 were high-volume hospitals (> 50 RLTs/year) which indicates a centralization of this therapy. In a previous study, we were able to show the correlation of caseload volume and perioperative mortality [17].

### Current trends and future situation

While analyzing our created maps, we observed that ^177^Lu-PSMA RLTs is been offered in all major cities across Germany in 2020. The present analysis showed that in 2020, roughly one-third of nuclear medicine departments performed more than 100 ^177^Lu-PSMA RLTs. Comparing to the results from 2016 where 16% of nuclear medicine departments performed >100 ^177^Lu-PSMA RLTs, we observed a further tendency towards centralization of the therapy. Until now, ^177^Lu-PSMA RLT was only performed in patients with mCRPC who exhausted the approved treatment regimens for mCRPC [25]. First clinical trials focused on ^177^Lu-PSMA RLT in mCRPC, since the response to chemotherapy and systemic therapies is usually limited in these patients and an urgent need for durable treatment options was needed [26].

Since the treatment’s results are very promising, several studies are now investigating ^177^Lu-PSMA RLT for other indications. Privé et al. published the first outcomes of a prospective pilot study with ^177^Lu-PSMA-617 treatment in low-volume hormone-sensitive metastatic PCa [27]. Besides maintenance of a good quality of life, half of the patients showed a PSA response of more than 50% [27]. Recently, the authors gave an update and showed that the median long-term follow-up of the cohort was 28 months (range 11–39 months) with the median progression-free survival of 11 months (range 4–39 months) [28]. Currently, it can be assumed that the expected EMA approval later this year will further accelerate the momentum of ^177^Lu-PSMA RLT and lead to an increase of case load. Future studies should provide an international comparison of the contemporary trend of ^177^Lu-PSMA RLT.

### Limitations

We acknowledge some limitations of our study. First, the German hospitals’ quality reports and the Destatis database lack clinical information such as Gleason Score or PSA values. Further, the quality reports may be subject to documentation errors since they are prepared by the hospitals during routine care [17]. Also, for methodological reasons, we are unable to record therapies that are not billed as inpatient treatments, e.g., studies. Initially, we planned a comparison with the multicenter WARMTH study recording routine treatment to further validate our analysis of billing data [29]. However, our analysis showed that patients were only included selectively within the WARMTH trial and therefore this study was not useful for validation of our results. We observed variation between centers in the coding of the procedure for reimbursement especially in the early phase of the newly in introduced therapy. This coding issue could have triggered underestimation of ^177^Lu-PSMA RLT therapies for mCRPC, but we were able to quantify this effect by supplementing analyses. In consequence, we performed a comparison with the less specific code “therapy with open radionuclides” (OPS code “8–530”) in combination with the diagnosis PCa (ICD code C.61) to quantify the difference as explained above. Thus, we provide the first population-based data analyzing ^177^Lu-PSMA RLT for mCRPC.

## Conclusion

In the present study, we observed a significant increase in ^177^Lu-PSMA RLT adoption for treatment of patients with mCRPC. This development is remarkable, because of outstanding formal EMA approval.

Interestingly, most of the therapies are performed at university hospitals with more than two-thirds of patients being older than 65 years. The expected approval by the EMA might be a further driver for the implementation of ^177^Lu-PSMA RLT in daily clinical routine.

## Supplementary Information

Below is the link to the electronic supplementary material.

Supplementary file1 (DOCX 19 kb)

## Data Availability

Data source: German research data center of the federal statistical office, DRG statistics 2006–2019, German “National Centre for Cancer Registry Data” (Robert Koch Institute, Berlin), own calculations. Data Availability Statement German hospital quality reports are publicly accessible. The datasets generated during and/or analyzed during the current study are available from the corresponding author on reasonable request.

## Abbreviations

^177^Lu: Lutetium-177
EMA: European Medicines Agency
FDA: Food and Drug Administration
ICD: International Statistical Classification of Diseases and Related Health Problems
LOS: Length of hospital stay
OPS: Operation and procedure codes
PET: Positron emission tomography
PSMA: Prostate-specific membrane antigen
RLT: Radioligand therapy
US: United States of America
