# Supplementary material for: Adoption of Lutetium-177 PSMA radioligand therapy for metastatic castration resistant prostate cancer: a total population analysis in Germany from 2016 to 2020
Source: Eur J Nucl Med Mol Imaging. 2023 Feb 24;50(7):2188–95. doi: 10.1007/s00259-023-06139-x (PMC10199877; doi:10.1007/s00259-023-06139-x)
Supplement: Supplementary file 1 — Supplementary file1 (DOCX 19 kb) [file 259_2023_6139_MOESM1_ESM.docx]

**Online Supplement**

**Table 1.** Lutetium-177 PSMA radioligand therapy cycles. (Source: German hospitals’ quality reports and caseload of two centers)

|  | **Quality report** | **Center**  **data** | **Number of patients** | **Quality report** | **Center data** | **Number of patients** | **Quality report** | **Center data** | **Number of patients** | **Quality report** | **Center data** | **Quality report** | **Center data** |
| --- | --- | --- | --- | --- | --- | --- | --- | --- | --- | --- | --- | --- | --- |
| **Marburg** | 40 | 39 | 19 | 42 | 43 | 20 | 43 | 41 | 24 | 56 | 52 | 64 | 63 |
| **Munich** | 1 | 163 |  | 126 | 192 |  | 284 | 300 |  | 244 | 348 | 253 | 352 |

**Fig. 1:** The green line indicates the total number of performed ^177^Lu-PSMA RLT per year. The blue columns represent the number of clinics providing the therapy. (Source: Destatis database).
